# Hematological Profile and Gametocyte Carriage in Malaria Patients from Southern Pakistan

**DOI:** 10.7759/cureus.4256

**Published:** 2019-03-15

**Authors:** Najia K Ghanchi, Mohammad Hassaan Khan, Muhammad Abdullah Arain, Mustafa Bin Ali Zubairi, Ahmed Raheem, Muhammad A Khan, Mohammad A Beg

**Affiliations:** 1 Pathology, Aga Khan University Hospital, Karachi, PAK; 2 Pathology and Laboratory Medicine, Aga Khan University Hospital, Karachi, PAK; 3 Pathology and Laboratory Medicine, Dow University of Health Sciences (DUHS), Karachi, PAK; 4 Community Health Sciences, Aga Khan University Hospital, Karachi, PAK

**Keywords:** gametocytes, plasmodium falciparum, plasmodium vivax, temperature

## Abstract

Background

Malarial infection is a major cause of concern, both worldwide and in Pakistan. Gametocytes are the sexual forms of the parasite that are essential for transmission. They fuse inside the mosquito to develop sporozoites. Gametocytes of the plasmodium parasites, which cause the infection, differentiate into male and female gametocytes. These gametocytes constitute the sexual stage of the malaria parasite and are essential in transmission of the disease from human to vector Anopheles. Gametocytes are affected by factors such as host immunity, drug treatment, reticulocytemia, anemia, low levels of asexual parasitemia and stress to the parasite. The aim of this study was to observe the hematological parameters, age and gametocyte carriage in an area of seasonal malaria transmission.

Methods

The study was conducted at Aga Khan University Hospital (AKUH) Laboratory over the period of one year and 294 patients with uncomplicated malaria were recruited. Patients infected with Plasmodium falciparum (P. falciparum) or Plasmodium vivax (P. vivax) malaria and no co-morbidities were included in the study.

Results

Gametocytemia was highest during the period of July to November, with P. vivax, 267 (90.8%), predominating compared to P. falciparum, 27 (9.2%). P. vivax gametocytes were observed from May to October and P. falciparum gametocytes were observed from July to December. Low hemoglobin in females and low platelet levels were observed. The mean platelet count was significantly lower in cases of P. vivax having gametocytes compared to P. falciparum with gametocytes. Higher parasitic index was associated with lower platelet count. The most significantly altered parameters were hemoglobin, hematocrit, white blood cell (WBC), and platelet count. Hemoglobin and platelets were significantly lower during the malaria season in study participants, both male and female.

Conclusion

In conclusion, infection with P. falciparum and P. vivax modulates significant changes in hematological parameters in populations living in malaria endemic regions. In the malaria season males were more frequently affected by malaria with thrombocytopenia. Gametocyte carriage remains unaffected by seasonal changes thus ensuring parasite transmission during the dry season.

## Introduction

Malaria is one of the leading causes of morbidity and mortality worldwide, with nearly half of the world population classified as ‘at risk’ in 2015. According to the World Health Organization (WHO), there were around 212 million malaria cases reported and an estimated 490,000 deaths caused by malaria in 2015 worldwide [1]. Pakistan is one of the countries affected, with 177 million out of the 185 million population being at risk, along with 0.9 million presumed and confirmed cases every year. Malaria transmission is seasonal and coincides with the monsoon season. Plasmodium falciparum (P. falciparum) and Plasmodium vivax (P. vivax) coexist in Pakistan and transmission is highest from July to November. Most of the Plasmodia after infecting the host undergo asexual replication, but a proportion differentiates into gametocytes. Gametocytes constitute the sexual stages of the malaria parasite and are essential in the transmission of the disease from human to its vector female Anopheles mosquito [2].

Gametocytes of P. falciparum are sequestered away from the peripheral circulation for maturation, and evidence has been presented for the development of gametocytes in the hematopoietic system of the human bone marrow [3]. It has also been postulated that the bone marrow could be a niche for gametocyte production and maturation and/or a reservoir in P. vivax infections. Thus, gametocytes appear in the blood stream seven to 15 days after, for P. falciparum, but much sooner, for P. vivax, after the initial appearance of asexual stage parasites [4].

While there is much information about the asexual forms of the parasite, not much is known of the sexual forms and the process of gametocytogenesis. Knowledge of factors inducing or affecting gametocytogenesis/gametogenesis may help facilitate actions which could disrupt the malaria transmission cycle. The conversion of asexual to sexual stages depends upon factors such as host immunity, drug treatment, presence of reticulocytes (in vitro), anemia, low levels of asexual parasitemia and stress to the parasite [5]. However, the stress may not be essential for gametocytogenesis, shown by the commitment to sexual development at early infection stages [6].

Presence of anemia is identified as a risk factor for gametocytaemia [7]. Factors that decrease the survival of asexual forms of the parasite, can lead to an increase in density of sexual forms. Anemia may lead to a greater chance of gametocyte formation [8]. Anemia may also lead to increased reticulocyte levels in the blood and reticulocyte-rich blood has been found to promote enhanced gametocyte formation in vitro [9-10].

Host immunity also plays a significant part in gametocyte production. Gametocytogenesis was induced in vitro for P. falciparum using lymphocytes and serum, acquired from P. falciparum-infected children [11]. Therefore, the host immunity not only increased gametocyte production directly, but also indirectly, by killing asexual stages [12].

Gametocyte carriage has also been shown to differ in different age groups and seasons. Gametocytemia is reported to be greater in the younger age groups as compared to the older, especially those aged <16 years [13]. Increased gametocyte carriage was observed during the dry season in patients residing on the Thai-Burmese border [14]. Furthermore, the continuous use of chloroquine (CQ) and sulphadoxine-pyrimethamine monotherapy in resistant infections results in increased gametocytemia and hence, increased transmission [15-16].

This study aims to document and compare the changes in hematological parameters of the malaria-infected patients, along with the variations in gametocyte carriage and malaria transmission during different seasons. This can help in a better understanding of malaria transmission and prognosis and according to a study conducted, these hematological variations when used in combination with other clinical and microscopy methods can improve malaria diagnosis and treatment [17].

In this study the changes in the hematological parameters, age and seasonal variation of gametocyte carriage were observed in an endemic area.

## Materials and methods

The study was conducted at Aga Khan University Hospital (AKUH) Laboratory, Karachi, Pakistan. A total of 294 patients infected with P. falciparum or P. vivax malaria were included. Patients who were malaria negative and refused to consent were excluded.

The study was approved by the Ethical Review Committee of Aga Khan University Hospital and conducted in accordance with the Good Clinical Practice of Declaration of Helsinki. Informed consent was obtained from enrolled patients.

Peripheral blood (1 ml) was obtained from the participants for detection of the malaria parasite. Thick and thin smears were prepared from each patient’s blood, and Giemsa staining performed, to quantify and identify the species of the parasite respectively. The malaria positive blood films were reviewed by an expert. Parasitemia was calculated using 100x oil immersion microscopy. The parasites were counted against 200 white blood cells (WBCs) and the parasite density was calculated with the help of the total leukocyte count (TLC). Participants were stratified into three groups based on age less than 15 years, 16-40 years and greater than 40 years.

Data was analyzed using SPSS Statistics for Windows, Version 21.0 (IBM Corp., Armonk, NY, USA). Arithmetical means and medians were calculated, where applicable, for all continuous baseline variables. Mann-Whitney U test and ANOVA test, if the assumption was found, then Kruskal-Wallis test were used to compare differences between different groups. Multivariate binary logistic regression was applied to compare the cause and effect among hematological parameters and demographics variable. p-value <= 0.05 was taken as significance at two-sided p-value.

## Results

In this study of 294 malaria-infected patients, 207 were males (70.4%) and 87 were females (29.6%). Gametocytemia with P. vivax, 267 (90.8%), was observed to predominate compared to P. falciparum, 27 (9.2%). P. vivax gametocytes were observed from May to October and P. falciparum gametocytes were noted from July to December.

The mean hemoglobin (Hb) and hematocrit (HCT) levels in females were observed to be low whereas, for males they were in the low-normal range (Table 1).

**Table 1:** Hematological parameters of patients infected with Plasmodium with respect to gender. * Normal values - Hemoglobin: 11.1-14.5 g/dl, hematocrit: 35.4-42%, total leukocyte count: 4-10 x 10^9^ L, platelets: 150-400 x 10^9^ L, red blood cell: 3.9-5.5 x 10^12^ L

Blood Parameters	Male (n = 207)	Female (n = 87)	p-value
	Mean ± SD	Mean ± SD	
Hemoglobin (g/dl)	11.85 ± 1.09	10.61 ± 0.58	<0.0001*
Hematocrit (%)	35.06 ± 3.21	31.84 ± 1.86	<0.0001*
Red blood cell count (x1012L)	4.26 ± 0.11	3.90 ± 0.30	<0.0001*
White blood cell count ( x109L)	6.53 ± 0.82	5.71 ± 0.31	<0.0001*
Platelets (x109L)	89.94 ± 5.64	103.9 ± 10.50	<0.0001*

On comparison of the different age groups, the Hb and HCT levels of the patients less than 15 years were found to be the most significantly affected (Table 2). A normal WBC count, along with thrombocytopenia, was seen in both male and female patients of all age groups. Excluding WBC count, all the other hematological parameters were significantly different between males and females (p < 0.0001).

**Table 2:** Hematological parameters of patients infected with Plasmodium with respect to different age groups. * Normal values - Hemoglobin (Hb): 11.1-14.5 g/dl, hematocrit (HCT): 35.4-42%, total leukocyte count: 4-10 x 10^9^ L, platelets: 150-400 x 10 ^9^ L, red blood cell (RBC): 3.9-5.5 x 10^12^ L

Hematological Parameters	<=15 Years (n = 36)	16-40 Years (n = 145)	>40 Years (n = 113)	Total (n = 294)	p-value
Hb	10.28 ± 2.15	12.41 ± 2.18	11.71 ± 2.34	11.86 ± 2.34	<0.001*
White blood cell (WBC)	5.82 ± 3.07	5.85 ± 2.37	6.92 ± 3.97	6.26 ± 3.2	0.018*
HCT	30.65 ± 6.02	36.77 ± 6.3	34.77 ± 6.93	35.19 ± 6.8	<0.001*
Platelets	96.5 (114.5-49.5)	82.5 (120.2-53)	86 (127-48.5)	85 (120-50.5)	0.933
RBC	3.98 ± 0.8	4.34 ± 0.75	4.06 ± 0.85	4.19 ± 0.81	0.005*

There was a significant difference in all hematological parameters between P. falciparum and P. vivax, except for the platelet levels. The HCT (p = 0.000), Hb (p = 0.000) and red blood cell (RBC) (p = 0.000) levels were all higher in P. vivax-infected patients while the WBC (p = 0.000) and platelet (p = 0.090) levels were greater in those suffering from P. falciparum infection (Table 3). Binary logistic regression analysis was performed between gametocytemia and independent variables such as RBC, platelet and Hb. RBC count were significantly lower with higher gametocytemia odd ratio 0.467 (0.251-0.867, P = 0.016*), male gender were more significantly 12.6 times gametocytaemia higher in Plasmodium vivax as compared to female subjects (p-value < 0.001*) while other parameters were not significantly associated with gametocytemia. Univariate analysis revealed that gametocyte carriage decreased with age, the proportion of gametocyte positive patients was highest in the younger age groups and decreased rapidly with age.

**Table 3:** Hematological parameter of patients infected with P. falciparum and P. vivax. * Normal values - Hemoglobin: 11.1-14.5 g/dl, hematocrit: 35.4-42%, total leukocyte count: 4-10 x 10^9^ L, platelets: 150-400 x 10^9^ L; red blood cell: 3.9-5.5 x 10^12^ L

Hematological Parameters	P. falciparum (n = 27)	P. vivax (n = 267)	p-value*
	Mean ± SD	Mean ± SD	
Hemoglobin (g/dl)	8.54 ± 1.25	12.19 ± 2.15	<0.001
Hematocrit (%)	24.49 ± 3.6	36.22 ± 6.16	<0.001
Red blood cell count (x1012L)	2.90 ± 0.42	4.32 ± 0.72	<0.001
White blood cell count (x109L)	9.52 ± 5.7	5.93 ± 2.6	<0.001
Platelets (x109L)	117.89 ± 138	94.09 ± 58	0.09*

Gametocyte density for P. vivax peaked earlier, around July-August, while that of P. falciparum came later, around September-October. On the other hand, the mean Hb, HCT, RBC and WBC levels were found to be similar in both the rainy and dry seasons but, the mean platelet levels were lower in the rainy season as compared to the dry season (Table 4).

**Table 4:** Effects of seasonal variation in hematological parameter of patients with gametocytaemia. * Normal values - Hemoglobin: 11.1-14.5 g/dl, hematocrit: 35.4-42%, total leukocyte count: 4-10 x 10^9^ L, platelets: 150-400 x 10^9^ L, red blood cell: 3.9-5.5 x 10^12^ L

Blood Parameters	Rainy season (n = 49)	Dry season (n = 245)	p-value*
	Mean ± SD	Mean ± SD	
Hemoglobin (g/dl)	8.54 ± 1.25	12.19 ± 2.15	0.8096
Hematocrit (%)	24.49 ± 3.6	36.22 ± 6.16	0.9918
Red blood cell count (x1012 L)	4.22 ± 0.82	4.06 ± 0.77	0.1655
White blood cell count (x109 L)	6.14 ± 2.87	6.70 ± 4.20	0.2191
Platelets (x109 L)	94.10 ± 68.62	104.24 ± 72.26	0.3049

**Figure 1:**
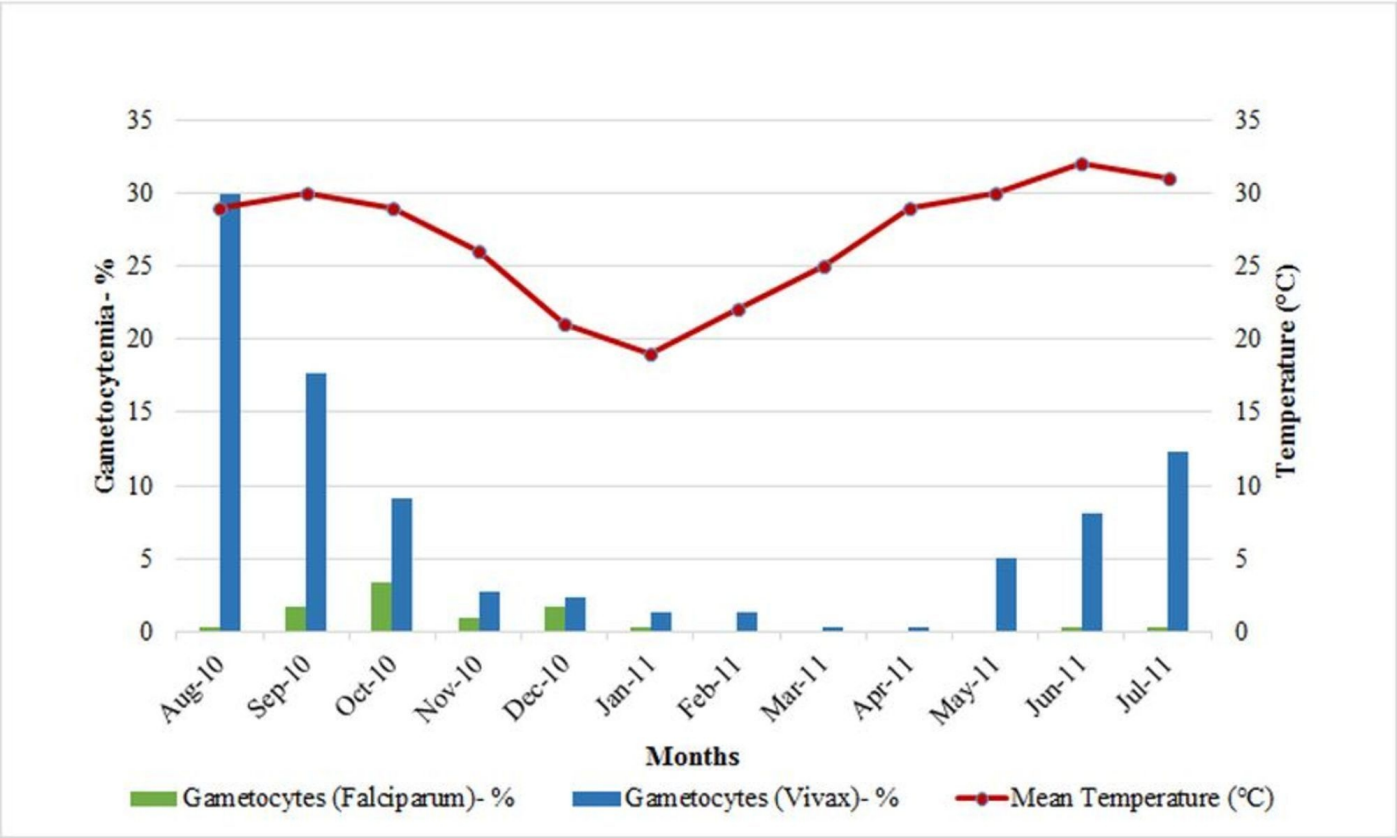
Monthly variation in gametocytes carriage and its association with rising temperatures.

Bar charts represent gametocyte carriage of both P. falciparum and P. vivax detected in over a year period. Percent gametocytemia detected monthly are indicated. Average monthly temperatures recorded are plotted and indicated as red line. High gametocytes carriage was noted during malaria season from June to October when temperatures were higher (above 30°C). Gametocytemia was seen to increase as the temperature increased and was high from May to November as shown in Figure 1.

## Discussion

The two most common malaria species, Plasmodium falciparum (P. falciparum) and Plasmodium vivax (P. vivax), have overlapping areas of distribution in Pakistan. Even though there is a higher incidence of P. falciparum infections throughout the world, our study showed a higher incidence of P. vivax species (90.8%) compared to P. falciparum (9.2%). These findings are consistent with the infecting patterns of this region [18-19]. We have also reconfirmed that gametocyte carriage was 4.8 times more common in vivax vs. falciparum patients [6]. Presence of gametocytes (P. vivax and P. falciparum) was seen in patients and analyzed according to their age. Univariate analysis revealed gametocyte carriage decreased with age, the proportion of gametocyte positive patients was highest in the younger age groups and decreased rapidly with age.

In this study, the changes in hematological parameters of the malaria-infected patients were observed. The platelet counts were found to be significantly low in all age groups, for both genders. On the other hand, the female patients were diagnosed with anemia. Anemia was common in female patients compared to male (p-value < 0.001*).

The majority of the Pakistani population suffers from anemia, especially iron deficiency anemia, and all age groups, from infants to elderly, are affected [20]. Anemia can be attributed to the destruction of red blood cells, splenic removal of parasitized and non-parasitized RBCs and dyserythropoiesis and engulfment in reticulo-endothelial system [21]. Anemia due to malaria is indicated by the destruction of both infected and uninfected RBCs, a reduction in erythrocyte precursors and inhibition of erythropoiesis eventually leading to severe malaria or death in patients. This is commonly seen in infections by P. falciparum species. Hemozoin is believed to play a major part in this by having an influence on the extrinsic apoptotic pathway of erythroid cells [22]. On the other hand, thrombocytopenia is a common laboratory observation in malaria patients and the exact etiology is not very well understood. The popular postulations suggest that macrophage-mediated immunologic lysis, splenic sequestration, ultra-structural changes and dyspoietic development in the bone marrow could be the likely causes [23]. Severe disease could also lead to disseminated intravascular coagulation (DIC) which would cause increased consumption of platelets causing thrombocytopenia in the process.

There have been different observations regarding the changes in WBC count due to malaria. A study conducted in Kenya stated that malaria causes leukocytosis, and this was associated with a worse prognosis [24]. Whereas, in another study near the Thailand-Myanmar border, the WBC count in the malaria-infected patients was seen to increase [25]. In our study, the WBC count of the patients was mostly in the normal range. As a result, we advise that the possibility of a normal WBC count in a malaria-infected patient along with thrombocytopenia and anemia should always be taken into consideration.

Malaria incidence usually has a seasonal repetition because the life span, population and rate of survival of the Anopheles mosquito depend upon climatological elements. Pakistan is a mesoendemic region which means there is a regular seasonal transmission which occurs under normal rainfall conditions [26]. In this study, the highest rate of gametocytemia was seen during the rainy season in the second half of the year. This time period lies in the high transmission season for malaria infectivity for Pakistan and corresponds to high temperatures and monsoon rainfall in the region, both factors that facilitate infection transmission. However, during the dry season a reservoir of gametocytes persists. P. falciparum gametocytes may persist for three to six weeks after the cure of infection [27] and analysis of a study revealed that P. vivax gametocyte appeared for up to 63 days after enrollment for treatment [28]. Hence, cases of gametocyte carriage have been reported from December to February facilitating malaria transmission. Gametocytaemia persists throughout the dry season (March-May).

Therefore, strict preventive measures should be taken in the monsoon season, when gametocytemia is at its peak, to drastically reduce the incidence of malaria.

This study also indicates a greater percentage of malaria infections in males than in females. In the Pakistani society, males are more exposed to outdoor activities, thus they are susceptible to mosquito bites. Karachi, where this study was conducted, is an industrial city and the waste water can provide breeding grounds for mosquito larvae, predisposing males to malaria infection [29-30].

Malaria causes changes in hematological parameters which are not always consistent. Anemia and thrombocytopenia are the two most common hematological observations with malaria but the WBC count may vary. Malaria transmission is greatest during the monsoon season but malaria infections in dry seasons can also be seen and it is essential to take preventive measures in order to reduce malaria incidence. One of the limitations of this study was that we only included hospitalized patients and sample size was relatively smaller.

## Conclusions

In conclusion, infection with P. falciparum and P. vivax modulates significant changes in hematological parameters in populations living in malaria endemic regions. The mean platelet count was significantly lower in cases of P. vivax having gametocytes compared to P. falciparum with gametocytes. Higher parasitic index was associated with lower platelet count. The most significantly altered parameters were hemoglobin, hematocrit, WBC, and platelet count. Hemoglobin and platelets were significantly lower during the malaria season in study participants, both male and female. In the malaria season, males were more frequently affected by malaria with thrombocytopenia. Gametocyte carriage varies seasonally and may persist at low levels during the dry season.
